# Effect of Pulsed Electric Field Treatment on the Protein, Digestibility, and Physicochemical Properties of Starch Granules in Wheat Flour

**DOI:** 10.3390/polym15204087

**Published:** 2023-10-14

**Authors:** Piyada Achayuthakan, Rungtiwa Wongsagonsup, Jiratthitikan Sriprablom, Manop Suphantharika, Panich Intra

**Affiliations:** 1 Food Industrial Microbiology and Bioinnovation Program, Faculty of Science and Technology, Suan Sunandha Rajabhat University, Dusit, Bangkok 10300, Thailand; 2 Food and Nutrition Academic and Research Cluster, Institute of Nutrition, Mahidol University, Nakhon Pathom 73170, Thailand; 3 Department of Biotechnology, Faculty of Science, Mahidol University, Bangkok 10400, Thailand; 4 Research Unit of Applied Electric Field in Engineering (RUEE), College of Integrated Science and Technology, Rajamangala University of Technology Lanna, Chiang Mai 50220, Thailand

**Keywords:** pulsed electric field, wheat flour, pasting properties, digestibility, confocal laser scanning microscopy

## Abstract

The effect of pulsed electric field (PEF) treatment depends mainly on the electric field strength and treatment time. In this study, wheat flour–water suspensions were treated with PEF at an electric field strength of 3 kV/cm for 0 to 1400 pulses to obtain a specific energy input of 0 to 656 kJ/kg. The effect of PEF on the removal or unfolding of proteins from the starch surface, digestibility, starch granule structure, and physicochemical properties of wheat flour was studied. The removal of proteins from the surface and the damage to the internal structure of wheat starch granules after PEF treatment was detected by confocal laser scanning microscopy (CLSM) and FTIR. The damage of the PEF-treated wheat starch granules was observed by scanning electron microscopy (SEM). From CLSM results, penetration of dextran (Mw 10,000 Da) into starch granules of wheat flour was dependent on the energy input of PEF. The high the energy input showed the intense penetration of the biopolymer. The benefits of the accessibility of biopolymer in starch granules are to increase enzyme digestion, especially rapidly digestible starch (RDS). The RDS of wheat flour treated with PEF at 656 kJ/kg was 41.72%, whereas the RDS of wheat flour control was 27.59%.

## 1. Introduction

Pulsed electric field (PEF) is an electroporation method that can create pores in the microbial cell membrane that allow the introduction of DNA fragments, chemicals, or drugs into the cell. A high-intensity pulsed electric field (10–80 kV/cm) is a practical non-thermal sterilization method due to its short time requirement and allows homogeneous treatment. Currently, customers are interested in minimizing food processing or using non-thermal methods that can preserve the freshness and flavor of products [1].

Generally, a PEF processing system consists of a high-voltage generator, an energy storage capacitor bank, a charging resistor, a discharging switch, a pulse controller, a treatment chamber (static or continuous), a raw material container, a finished product container, and control devices. The electrical energy from the power supply is collected in the capacitor and then discharged through the treatment chamber to create an electric field strength in the food product. Non-thermal processing at temperatures below 30–40 °C can avoid denaturation of foods containing proteins and vitamins. PEF technology can be applied to liquid, semi-solid, and solid foods, e.g., milk, orange juice, tomato paste, and fruit. PEF technology can extend the shelf life of food by breaking the cell membranes of foodborne pathogens and spoilage microorganisms, such as *Escherichia coli*, *Streptococcus faecalis*, *Bacillus subtilis*, *Streptococcus cremoris*, and *Micrococcus radiodurans* [2]. Sale and Hamilton [3,4] applied electric fields and determined the lethal effects on pathogenic bacteria by electroporation. They concluded that bacterial cells lost their membranes after treatment with PEF, resulting in cell death.

The principle of PEF treatment of microbial cell cultures and starch granules is the same. PEF can be used for electroporation by piercing the cell membrane, regardless of the size or shape of the cell [5]. However, the effects of PEF treatment on starch granules are more complex. PEF treatment changes the crystallinity of starch granules by destroying the crystalline structure [6]. The effects of PEF on starch granules depend on the morphology, crystalline structure (starch type A, B, or C), and botanical origin of the starch [7]. In addition, flour is composed of many components that are also affected by PEF treatment, such as proteins and lipids. The secondary structure of the protein associated with the oat starch granules was changed during PEF treatment [8]. The modification of starch by PEF techniques has already been extensively discussed in the literature [9,10,11].

PEF treatment with electric field strengths of 0.5 to 1 kV/cm and a treatment duration of 102–104 s enabled effective disintegration of cell membranes in beet and potato samples [12,13]. Native starches have been physically modified by PEF treatment to overcome their limited applications. PEF is an environmentally friendly treatment for starch modification [14]. PEF-modified starches not only have desirable functionalities, but are also classified as “clean label” ingredients [14]. Demand for these physically modified starches is increasing among consumers who prefer natural and healthy foods [11]. Factors affecting PEF treatment results include starch type and concentration, electrical conductivity, electric field strength, number of pulses, pulse width, pulse waveform, and treatment duration [15]. PEF treatment at high electric field strengths of 30–50 kV/cm was carried out to modify potato starch [16], corn starch [16,17], tapioca starch [18], and waxy rice starch [19], and at moderate electric field strengths of less than 10 kV/cm to modify glutinous rice grain [20], japonica rice starch [21], oat flour [22], and potatoes [23]. However, it is much more difficult and energy consuming to generate high PEF field strengths (30–50 kV/cm) than to treat materials at lower PEF field strengths for extended periods of time for industrial applications [20]. From the above literature, PEF generally reduced the molecular weight of starch molecules, the crystallinity of starch granules, the viscosity of starch during pasting, and the gelatinization temperatures and enthalpy, while increasing in vitro digestibility of starch, the extent depending on PEF treatment conditions. PEF treatment also facilitated enzymatic hydrolysis [24] and chemical modification [25] of starch.

Wheat (*Triticum aestivum* L.) flour is a versatile ingredient and is widely used in the production of homemade and almost all commercial baked goods (e.g., bread, cakes, and cookies) and pasta products (e.g., spaghetti and lasagna). It is unique for the presence of proteins that form a gluten network and give the wheat flour dough viscoelastic properties that allow the expansion of the dough and the storage of gas. However, the constant innovation in food products and their higher quality requirements have forced the food industry to search for flours with new specific functions and properties. Chemical, enzymatic, and/or physical modifications have been the alternatives to change the functionalities and properties of wheat flour [26].

Even though there are some reports on the effects of PEF treatment on the properties of wheat starch alone [7] and wheat gluten protein alone [27], to date, there are no reports on the effects of PEF treatment on the properties of wheat flour consisting of both starch and protein. We hypothesized that the interaction between the starch and non-starch components, such as proteins and lipids, plays an important role in the properties of wheat flour during PEF treatment. Therefore, the aim of this study was to investigate the effect of PEF treatment at a low electric field strength of 3 kV/cm, with different specific energy inputs ranging from 0 to 656 kJ/kg, on the protein, starch granules, digestibility, particle size distribution, and physicochemical properties of wheat flour.

## 2. Materials and Methods

### 2.1. Materials

Commercial refined all-purpose wheat (*Triticum aestivum* L.) flour with a protein content of 10.50–11.00% was obtained from United Flour Mill Public Company Ltd. (Bangkok, Thailand). Wheat gluten was purchased from Verasu company, Bangkok, Thailand. Ethanol was purchased from Sigma-Aldrich (Bangkok, Thailand) Company Ltd. The ethanol used in this experiment was of analytical grade. Distilled water was used to prepare the starch dispersion.

### 2.2. PEF Treatment of Wheat Flour

The PEF system consists of an AC 220 V supply line into 3 kV using a high-voltage transformer and then rectifies the AC high voltage into DC high voltage. A pulse controller delivers monopolar pulses of nearly rectangular shape with a constant pulse width of 10 μs and a pulse frequency of 1 Hz. The experiments were performed at ambient temperature. The treatment chamber was rectangular in shape (W × L × H: 4 × 40 × 32 cm) and consisted of two essentially parallel stainless steel plates connected on each side with electrical wires and sealed at the bottom with Teflon as an insulating spacer. The parallel plate treatment chamber with a gap of 40 mm was used in this study because the parallel plate electrodes provide uniform electric field distribution along the gap axes and electrode surfaces.

Wheat flour dispersion (10%) was placed in the batch treatment chamber at 15 °C and treated with PEF at 3 kV/cm for 0–1400 pulses, corresponding to a total specific energy input of 0 to 656 kJ/kg wheat flour. The PEF treatment was adjusted to a wide range from 0 to 656 kJ/kg to investigate the effects of specific energy input on the physical properties of wheat flour at a low electric field strength of 3 kV/cm. The sample temperature was measured after the treatment. The sample temperature did not exceed 35 °C after each treatment. The wheat flour dispersion was cooled in a water ice bath immediately after PEF treatment. The wheat flour was separated from the water by centrifugation, washed with 50% ethanol, and air dried on a tray. The dried wheat flour was ground into powder with a mill (IKA^®^ Tube Mill 100 control, IKA-Werke GmbH & Co. KG, Staufen im Breisgau, Germany) at a speed of 10,000 rpm for 1 min and sieved with a 200 mesh sieve.

### 2.3. Scanning Electron Microscopy (SEM)

Samples were mounted on stubs with double-sided tape, coated with AuPd for 3 min, and imaged in a JEOL JSM -IT300 SEM (JEOL USA Inc., Peabody, MA, USA) with an accelerating voltage of 5 kV, a working distance of 10 mm, an aperture of 3, and a probe current of 3 × 10^−11^. Micrographs were taken at 5000× magnification to visualize the surface properties. The digital images were taken with a resolution of 1280 × 960 and a dwell time of 160 s.

### 2.4. Confocal Laser Scanning Microscopy (CLSM)

Confocal laser scanning microscopy (CLSM) was carried out to visualize the microscopic distributions of protein and starch in the flour samples before and after PEF treatment using the method described by [28] with slight modifications. The native wheat flour (0 kJ/kg) and wheat flour treated with PEF at 188 and 656 kJ/kg were spread on a glass slide and stained with a mixed dye consisting of 0.01% fluorescein isothiocyanate (FITC) (Sigma-Aldrich, St. Louis, MO, USA) dissolved in 50% ethanol to label the starch and 0.01% rhodamine B (Sigma-Aldrich, MO, USA) dissolved in distilled water to label the protein, allowing simultaneous observation of starch (in green) and protein (in red) by CLSM. CLSM was also performed to investigate the extent of penetration of dextran molecules into the starch granules. The wheat flour samples were stained with 1% FITC-dextran (Mw = 10,000 Da) (MCE, MedChemExpress, Monmouth Junction, NJ, USA) according to the method described by [29]. All stained samples were incubated in the dark at room temperature for 1 h before viewing with CLSM. A drop of water was placed on the coverslip before imaging with a water immersion model. A 60× objective lens was used in this study. A Fluoview FV10i confocal laser scanning microscope (Olympus, Tokyo, Japan) with four diode lasers (405, 473, 559, and 635 nm) was used to image the wheat flour samples at excitation wavelengths of 473 and 561 nm for FITC and rhodamine B, respectively. The digital CLSM images were acquired using FV10i Fluoview software.

### 2.5. Pasting Properties

A rapid visco analyzer (RVA), model RVA-TecMaster (Perten Instruments, PerkinElmer Company, Segeltorp, Sweden), was used to determine the pasting properties of wheat flour before and after PEF treatment. The moisture content of wheat flour samples was determined using a moisture analyzer (Denver Instrument, model IR35, Bohemia, NY, USA). The moisture content of the wheat flour samples was 12–15% (*w*/*w*). Wheat flour was weighed (1.5 g, dry weight) in an aluminum RVA sample canister, and distilled water was added to obtain a total weight of 25 g (6% flour concentration). The standard 1 profile of the heating and cooling program was used in this study. Samples were held at 50 °C for 1 min, heated from 50 °C to 95 °C for 3 min 42 s, held at 95 °C for 2 min 30 s before decreasing to 50 °C for 3 min 48 s, and held at 50 °C for 2 min. The pasting temperature, peak, trough, breakdown, final, and setback viscosities were determined for wheat flour samples treated with PEF at specific energy inputs ranging from 0 to 656 kJ/kg. Thermocline for Windows (TCW3) software was used for operation and data analysis.

### 2.6. Differential Scanning Calorimetry (DSC)

The thermal properties of the native and PEF-treated wheat flours were analyzed using a DSC1 Star^e^ System differential scanning calorimeter (Mettler Toledo International Inc., Greifensee, Switzerland). Wheat flour (20 mg, 30% (*w*/*w*) concentration in distilled water) was hermetically sealed in a 40 μL aluminum pan. Samples were heated from 25 to 100 °C at a heating rate of 10 °C/min. An empty aluminum pan was used as a reference. The onset (*T*_o_), peak (*T*_p_), and conclusion (*T*_c_) gelatinization temperatures were recorded. The gelatinization enthalpy (Δ*H*) was analyzed based on the moisture-free and protein-free basis of the wheat flour according to [30]. STARe Evaluation Software was used to analyze and evaluate the data.

### 2.7. X-ray Diffraction (XRD)

X-ray diffraction analysis was performed using an X-ray diffractometer (model D8 Discover, Bruker AXS, Karlsruhe, Germany) equipped with a conventional copper target X-ray tube operating at 30 kV and 30 mA. The wheat flour samples treated with PEF at specific energy inputs of 0–656 kJ/kg were placed on the slides for X-ray scanning. Data were collected from 2θ of 5° to 40°. Crystallinity (%) was defined as the percentage ratio of the diffraction peak area to the total diffraction area. Native starch granules have a crystallinity between 15 and 45%. 

### 2.8. Fourier-Transform Infrared Spectrometer (FTIR)

Fourier-transform infrared (FTIR) measurements of wheat gluten, the wheat flour control sample, and wheat flour treated with PEF at 656 kJ/kg specific energy inputs were carried out by using a Spectrum One FT-IR spectrometer (Perkin Elmer, Shelton, CT, USA) equipped with a MCT detector. The samples and chromatographic pure KBr (Fisher Chemical, Pittsburgh, PA, USA) were mixed in a ratio of 1:100, ground, and pressed into 20 mg thin slices. The range of 0–2500 cm^−1^ was scanned at a resolution of 4 cm^−1^ to obtain the spectra. PerkinElmer FT-IR Spectrum 10 spectroscopy software was used to analyze the Fourier infrared spectrum.

### 2.9. Particle Size Distribution

The starch particle size distribution was detected using a Mastersizer 2000 instrument (Malvern Instruments ltd., Worcestershire, UK). The samples were diluted with deionized water and stirred at 2000 rpm. The refractive index of the starch samples was 1.53.

### 2.10. In Vitro Starch Digestibility of Flour

The digestibility of starch in the flour samples studied was determined in uncooked states according to the procedures of Englyst et al. [31] with slight modifications. The flours (0.80 g, dry basis), guar gum (0.05 g), and sodium acetate buffer (0.25 M, pH 5.2) (20 mL) were mixed with an enzyme solution of pancreatin and amyloglucosidase (5 mL) thoroughly. The mixture was incubated at 37 °C in a water bath with shaking for 2 h. During incubation, aliquots (0.25 mL) of the mixture were taken at different points in time (20, 60, 90, and 120 min). These aliquots were then mixed with ethanol (66% *v*/*v*, 10 mL) to terminate the enzymatic reaction and then centrifuged at 1500× *g* for 10 min. The supernatant was used to analyze the amount of glucose released using the GOPOD assay kit (Megazyme International Ireland Ltd., Wicklow, Ireland). The percentage of starch hydrolysis at different hydrolysis times and starch fractions (RDS, SDS, and RS) were calculated as follows:

Starch hydrolysis in each hydrolysis time (%) = (G_ht_/TS) × 0.9 × 100

  RDS (%) = (G20/TS) × 0.9 × 100

      SDS (%) = [(G120 − G20)/TS] × 0.9 × 100

RS (%) = 100 − (RDS + SDS)

where G_ht_ is the glucose content released in each hydrolysis time; G20 and G120 are the glucose content released at 20 and 120 min of hydrolysis, respectively; and TS is total starch content.

### 2.11. Statistical Analysis

All sample analyses were performed in triplicate, and statistical analysis was performed using SPSS v. 22 (SAS Institute Inc. Cary, NC, USA). Duncan’s multiple range test was used to estimate significant differences among means at a probability level of 5% (*p* ≤ 0.05).

## 3. Results

### 3.1. Scanning Electron Microscopy (SEM)

**Figure 1:**
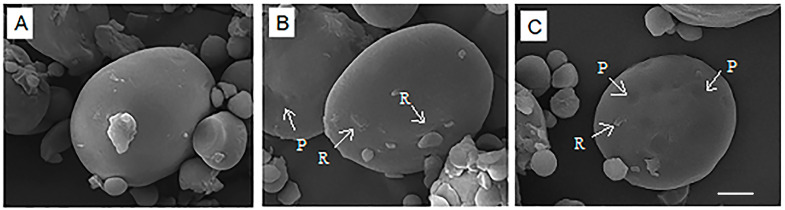
Scanning electron microscopy (SEM) images of native wheat flour (**A**) and PEF-treated wheat flours at specific energy inputs of 188 (**B**) and 656 kJ/kg (**C**). The magnification of all figures was 3000×. The symbol P stands for pore and R for roughness. Scale bar was 5 μm.

**Figure 2:**
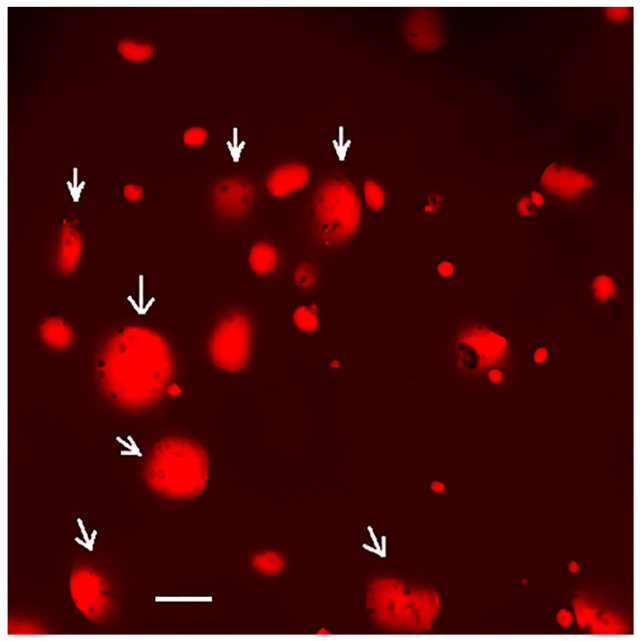
Confocal laser scanning micrographs focused on the surface of wheat flour treated with PEF at 656 kJ/kg and stained with rhodamine B. Scale bar was 20 μm. White arrows showed shallow pores on surface of many starch granules.

The SEM micrographs of native and various PEF-treated wheat flours are shown in Figure 1. The surface morphology of the native wheat starch granules was smooth and round with irregular shape and size (Figure 1A). After PEF treatment at 188 kJ/kg, most of the starch granules remained intact. However, some roughness or damage had developed on the surface, as shown by the white arrows (Figure 1B). PEF treatment at 656 kJ/kg resulted in granules with higher roughness or damage, and some shallow pores and cavities were found on the surfaces of individual granules, as shown by the white arrows (Figure 1C) and Figure 2 confocal laser scanning micrographs. It was likely that the damage to the outer part of the starch granule allowed the inner part of the granule to effectively absorb water and swell [17]. Maniglia et al. [14] showed the fractures of wheat starch after PEF treatment. Qiu et al. [20] studied the effect of PEF treatment at a field strength of 3 kV/cm for 50 to 300 pulses. They found that micropores were formed on the surface of PEF-treated rice grains, which increased the porosity of the grains. PEF creates cell membrane to form micropores, which increases the permeability of the cell membrane [32].

### 3.2. Confocal Laser Scanning Microscopy (CLSM)

**Figure 3:**
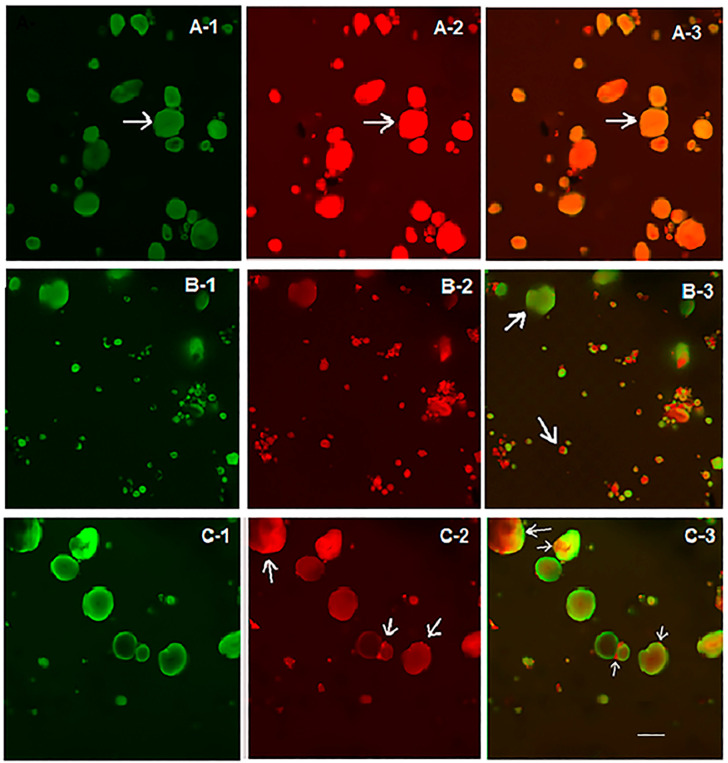
Confocal laser scanning micrographs of optical sections of native wheat flour (**A1**–**A3**) and PEF-treated wheat flours at specific energy inputs of 188 (**B1**–**B3**) and 656 kJ/kg (**C1**–**C3**). The flour samples were stained with FITC (green: (**A-1**,**B-1**,**C-1**)), rhodamine B (red; (**A-2**,**B-2**,**C-2**)) and FITC and rhodamine B (orange: (**A-3**,**B-3**,**C-3**)). Magnification was 60× for all samples. Scale bar was 20 μm.

CLSM with a fluorescent labeling technique using mixed dyes was used to localize gluten protein components and starch granules in wheat flour. FITC and rhodamine B were used to label starch granules (green) and gluten protein (red), respectively. The microscopic distribution of gluten protein and starch granules in the flour samples before and after PEF treatment is shown in Figure 3. In the control native wheat flour, all starch granules appeared reddish-orange, indicating complete coverage by proteins (Figure 3(A-3)), as indicated by white arrows. However, after PEF treatment at 188 kJ/kg, some starch granules appeared green and some reddish-orange (Figure 3(B-3)), while most starch granules appeared green and slightly red after PEF treatment at 656 kJ/kg (Figure 3(C-3)). These results clearly show that surface proteins were largely extracted from the surface of starch granules during PEF treatment, with the extent increasing with increasing specific energy input. It is well recognized that PEF is an effective technique for extracting proteins from starch [33,34]. Removal of proteins from the surface of starch granules could affect the physical and functional properties of wheat flour. The effects of PEF treatment on the physical properties of starch and the secondary structures of proteins in oat flour were reported by Duque et al. [8]. Xu et al. [35] proposed to improve the quality of wheat chip by using protease to remove proteins from starch granules and weaken the gluten strength of the dough. They found that wheat flour treated with protease reduced the hardness of wheat chip. The partial removal of proteins in wheat flour by PEF treatment could be used as a modification of wheat flour in baked goods.

**Figure 4:**
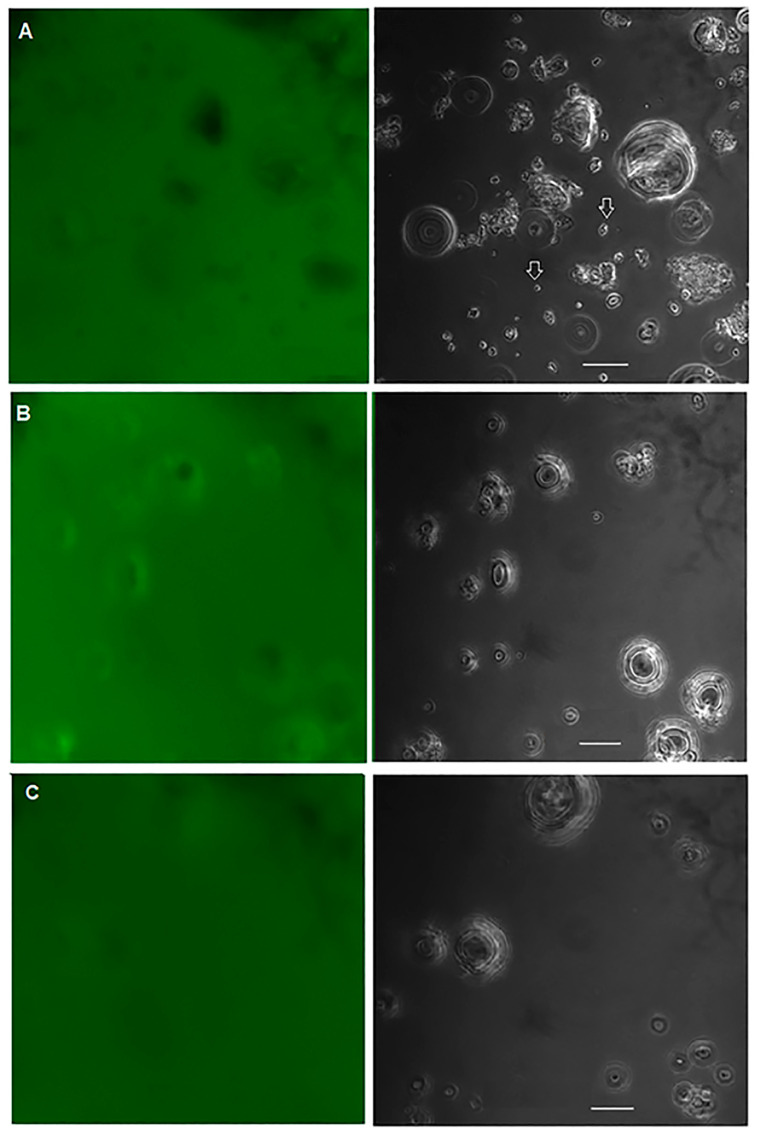
Confocal laser scanning micrographs of optical sections of native wheat flour (**A**) and PEF-treated wheat flours at specific energy inputs of 188 kJ/kg (**B**) and 656 kJ/kg (**C**); fluorescence (left) and bright field images (right). Wheat flour samples stained with FITC-dextran (Mw = 10,000 Da) were used to view the penetration of dextran into the starch granules. Scale bar was 20 μm.

The native wheat flour and the wheat flour treated with PEF at specific energy inputs of 188 and 656 kJ/kg were stained with FITC-dextran (Mw = 10,000 Da) to indirectly observe the destruction of the crystallinity of the starch structure after PEF treatment by CLSM. Our results showed that FITC-dextran barely penetrated native wheat starch granules (Figure 4A). Moreover, the small granules which were pointed by white arrows showed fully penetration of FITC-dextran. Black spots in the center of the starch granules indicated the area of the starch granules where FITC-dextran could not penetrate. After PEF treatment at 188 kJ/kg, FITC-dextran penetrated some starch granules, while other granules were only partially penetrated, indicating the destruction of the crystallinity of some starch granules (Figure 4B). After PEF treatment at 656 kJ/kg, almost all starch granules were thoroughly penetrated by FITC-dextran (Figure 4C). This is the first scientific article using CLSM to visualize the effects of PEF on structural changes within starch granules. Using CLSM observations, Yu et al. [24] showed that PEF modification improves the sensitivity of starch granules to enzymes. Our results agree well with those of Qiu et al. [20], who showed an increase in the porosity of the rice grain from 7.3% to 9.8% and an increase in the degree of swelling after PEF treatment at a field strength of 3 kV/cm for 50 to 300 pulses. The increase in porosity of starch granules could improve the penetration of FITC-dextran into the granules.

In addition, wheat starch granules have surface pores and channels that extend into the interior of the granules [36]. Removal of surface proteins by PEF treatment (Figure 3) exposed the pores and channels of starch granules to the environment and facilitated penetration of FITC-dextran into the granule matrices. Ma et al. [37] also reported accelerated diffusion of dextran probes into internal cavities and matrices of rice starch granules after removal of surface proteins from the granules. Starch pores and channels could open widely during starch gelatinization or after PEF treatment due to the destruction of the crystalline region.

### 3.3. Pasting Properties

The pasting properties of the native and the various PEF-treated wheat flours are shown in Table 1. Peak viscosity decreased significantly (*p* ≤ 0.05) with increasing specific energy input. This could be due to the damage of the surface structure of the wheat starch granules, as shown in the SEM images (Figure 1) [6,20,21]. The trough and breakdown viscosities also decreased with increasing specific energy input, indicating an improvement in the hot paste stability of the PEF-modified wheat flour. The viscosity of wheat flour treated with PEF at high specific energy input was lower than the viscosity of wheat flour treated with PEF at low specific energy input or the control. The lower values of breakdown viscosity indicate that the PEF-treated wheat flour is more resistant to mechanical and thermal treatment during cooking. Therefore, these modified flours could be used in thermally processed foods [6]. The final and setback viscosities decreased significantly (*p* ≤ 0.05) with increasing specific energy input, indicating an improvement in the cold paste stability of the PEF-modified wheat flour. The setback viscosity reflects the retrogradation tendency of amylose in the starch paste and is related to the texture of various food products. Therefore, PEF treatment is considered desirable for improving product quality since retrogradation usually causes quality deterioration characterized by syneresis or increased hardness [8,21]. Compared to the native wheat flour, the pasting temperature did not change until the specific energy input of 375 kJ/kg and then increased significantly (*p* ≤ 0.05) at 469 kJ/kg, remaining almost constant until 656 kJ/kg. At an electric field strength of 3 kV/cm, Qiu et al. [20] found that the pasting temperature of glutinous rice flour remained almost unchanged when the number of PEF pulses was increased from 50 to 300, which is consistent with our results. This could be due to the presence of a considerable amount of proteins that could maintain the onset of swelling of the starch granules at the same temperature. However, these results contradict the findings of Duque et al. [8], who found that the pasting temperature of PEF-treated oat flour significantly decreased with increasing specific energy input from 48 to 484 kJ/kg at electric field strengths of 2.0 and 4.4 kV/cm. This discrepancy could be attributed to the different types of flour and PEF conditions.

### 3.4. Thermal Properties

The onset (*T*_o_), peak (*T*_p_), and conclusion (*T*_c_) gelatinization temperatures; the gelatinization temperature range (Δ*T = T*_c_ − *T*_o_); and the gelatinization enthalpy (Δ*H*) of the native and the different PEF-treated wheat flours calculated from the DSC measurements are listed in Table 2. Zaidul et al. [38] reported that the values of *T*_o_, *T*_p_, and Δ*H* obtained from DSC analysis of 30% native wheat flour suspension were 57.0 °C, 62.3 °C, and 5.3 kJ/kg, respectively, which agrees well with our results. In addition, the presence of gluten protein in native wheat flour was reported to decrease gelatinization enthalpy and increase gelatinization temperatures of wheat starch. This could be due to the fact that the gluten protein and wheat starch compete for water, resulting in less water available for starch gelatinization [39]. Eliasson et al. [30] reported that Δ*H* of the different types of wheat flour ranged from 8.56 to 9.51 kJ/kg, whereas the Δ*H* of the control native wheat flour in this study was 4.24 kJ/kg. In addition, Tiga et al. [40] found that *T*_o_, *T*_p_, and Δ*H* of wheat flour were 57.07 °C, 64.12 °C, and 0.93 kJ/kg, respectively.

In this study, *T*_p_, *T*_c_, Δ*T*, and Δ*H* values of the PEF-treated wheat flour significantly (*p* ≤ 0.05) decreased with increasing specific energy input, while *T*_o_ remained almost unchanged. The gelatinization temperatures and enthalpy could be related to the degree of crystallinity and the amylopectin structure of the starch granules [20]. Han et al. [6] reported that gelatinization temperatures could be related to the degree of perfection of crystallites in starch granules, whereas gelatinization enthalpy could be related to the degree of crystallinity. PEF treatment with an appropriate input energy could change the internal structure of starch granules, resulting in lower attractive forces in the amorphous region and weaker intramolecular hydrogen bonds, so that gelatinization occurs at lower temperatures [20]. From the XRD analysis (Section 3.5), it appears that the PEF treatments with high specific energy input could destroy the crystalline regions of wheat starch granules and lead to a decrease in relative crystallinity. Therefore, during the gelatinization process, the water molecules can react more easily with the starch molecules in the crystalline region, which leads to a decrease in the Δ*H* values [16,18]. Han et al. [17] also reported decreases in *T*_o_, *T*_p_, and Δ*H* values of maize starch with increasing electric field strength and treatment duration, with electric field strength playing a dominant role in PEF treatments. Qiu et al. [20] reported significant reductions in *T*_o_ and Δ*H* values of glutinous rice flour when the number of PEF pulses was increased from 50 to 300 pulses at a field strength of 3 kV/cm. The gelatinization temperature range (Δ*T*) of wheat flour also decreased significantly (*p* ≤ 0.05) with increasing specific energy input. This is usually associated with increased crystallite homogeneity of starch granules. PEF treatment may have facilitated the fusion of weak crystallites, lowering the energy required for melting and ultimately increasing the interactions between the remaining crystallites [8]. Overall, our results suggest that PEF-treated wheat flour has high potential for use as a food ingredient due to its improved pasting and gelatinization properties, which can improve baking quality.

### 3.5. X-ray Diffraction (XRD)

**Figure 5:**
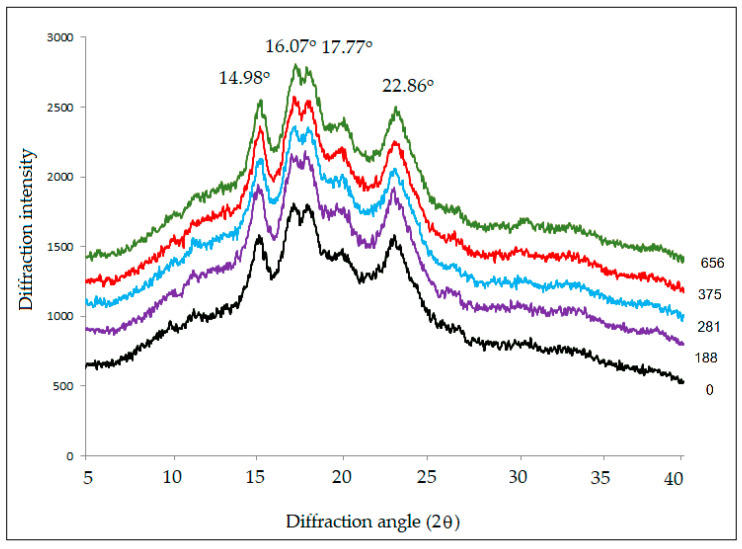
X-ray diffraction patterns of native and PEF-treated wheat flours at a field strength of 3 kV/cm with specific energy inputs between 188 and 656 kJ/kg.

The X-ray diffraction (XRD) patterns of native and various PEF-treated wheat flours are shown in Figure 5. The native wheat flour showed an A-type XRD pattern with four strong reflections at 2θ angles of 14.98°, 16.07°, 17.77°, and 22.86°. Relative crystallinity decreased slightly from 17.48% to 13.81% when specific energy input was increased from 0–656 kJ/kg. These results indicate that the crystalline structure of wheat flour was transformed into non-crystalline states after PEF treatment. According to many researchers [16,18,19,20,21,26], PEF treatment tends to destroy the crystalline regions of various native starch granules. Li et al. [7] reported slight variations in the relative crystallinity (i.e., slight increase at lower electric field strength and decrease at higher electric field strength) of starches with different crystalline types (A, B, and C) after PEF treatment in the range of 2.86–8.57 kV/cm. Hong et al. [25] found that the crystallinity of wheat starch decreased by 1.7% and 1.2% for A- and B-type starches, respectively, after PEF treatment. These results indicate that PEF tends to alter the structure of all starch types.

Abduh et al. [23] studied the effect of PEF treatment at electric field strengths of 0–1.1 kV/cm on potatoes. They found that PEF did not change the properties of potato starch extracted from PEF-treated potatoes. This could be attributed to the low electric field strengths compared with previous reports (30–50 kV/cm) [16,18]. It is likely that PEF treatment at a relatively low electric field strength of 3 kV/cm, which was applied to wheat flour in this study, could have little effect on the crystallinity of wheat flour.

### 3.6. Fourier-Transform Infrared Spectrometer (FTIR)

**Figure 6:**
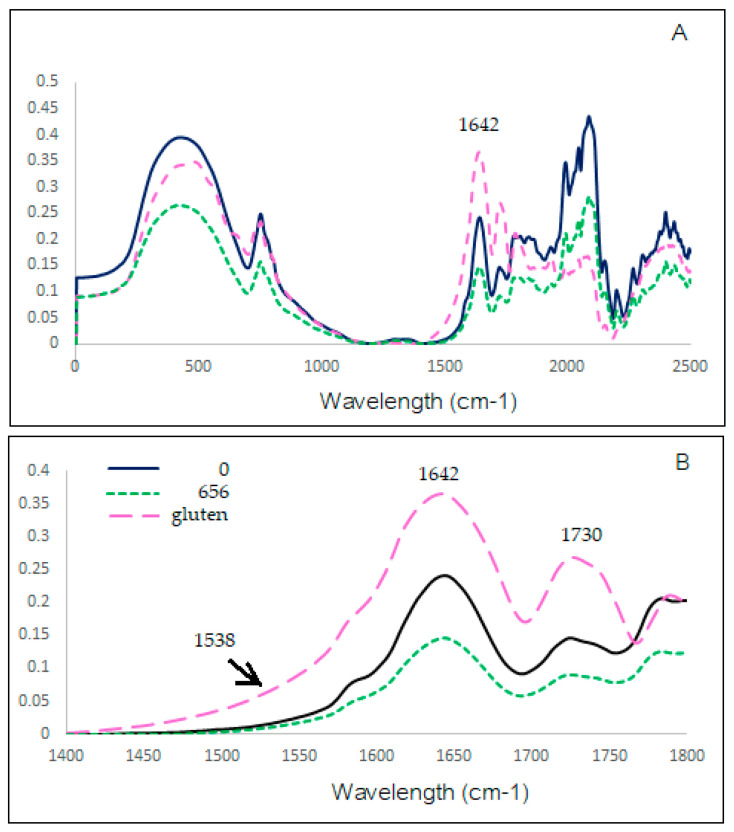
FTIR transmittance spectra of gluten (pink line), wheat flour control (black line), and treated with PEF at 656 kJ/kg (green line) 0–2500 spectra (**A**), 1400–1800 spectra (**B**).

The short-range ordered structure in starch was formed by the arrangement of adjacent starch chains that can be measured by using FTIR. From results in Table 3, the peaks at 995 cm^−1^ and 1047 cm^−1^ of FTIR spectra were related to the amount of crystalline, while the peak at 1022 cm^−1^ was related to the content of amorphous structures. The decrease of 1047/1022 and 995/1022 showed the decrease in the short-range molecular structure and the decrease in the double helix in the starch after PEF treatment, especially at high electric field strength [41]. The double helix tended to be loose and disordered after PEF treatment. The results were consistent with the decrease in enthalpy in wheat flour samples after PEF treatment in Table 2. In the FTIR spectrum, the peak at 1538 cm^−1^ was related to the formation of starch–protein complex by forming the C-N amide II band [42]. In this study, we have no peak at 1538 cm^−1^. However, the shoulder at 1538 cm^−1^ of wheat flour treated with PEF at 656 kJ/kg showed the lower intensity compared with the wheat flour control and gluten. That showed the decrease in the starch–protein interaction after PEF treatment. FTIT results of overlay spectra (Figure 6) of our study were consistent with the report of Zou et al. [42] that gluten showed a strong absorption band in the amide I (1600–1700 cm^−1^) region of FTIR. The wheat flour control sample and wheat flour treated with PEF at 656 kJ/kg showed the decrease in the gluten band at peak 1642 cm^−1^ after PEF treatment. Qiu et al. [20] concluded that PEF at 3 kV/cm was able to change the secondary structure of rice protein. Bands at the region of 1700 to 1200 cm^−1^ were reported to be related to protein and lipid components in the rice flour (Warren, et al. [43]). The peak at 1730 of wheat flour treated with PEF at 656 kJ/kg decreased compared with the wheat flour control.

### 3.7. Particle Size Distribution

**Figure 7:**
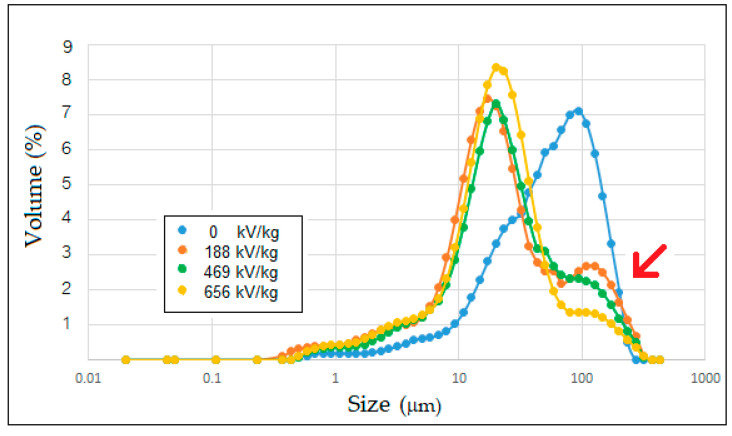
Particle size distribution of native and PEF-treated wheat flours at a field strength of 3 kV/cm with specific energy inputs between 188 and 656 kJ/kg.

Granule disruption of wheat starch granules was revealed in Figure 7. PEF treatment intensely decreased the particle size of wheat starch. The mean D (4,3) of particle size of the control was 71 μm. The means of the particle size of PEF-treated wheat starch granules at 188, 469, and 656 kV/kg were 46, 43, and 35 μm, respectively. The effect of PEF treatment was especially evident in large molecules showed with the red arrow in Figure 7. However, Maniglia, et al. [44] reported that PEF-treated wheat starch was a slight reduction of the medium-size molecules. For food application, Shen, et al. [45] reported that the starch particle size distribution could affect the noodle quality by changing the gluten network.

### 3.8. In Vitro Starch Digestibility

**Figure 8:**
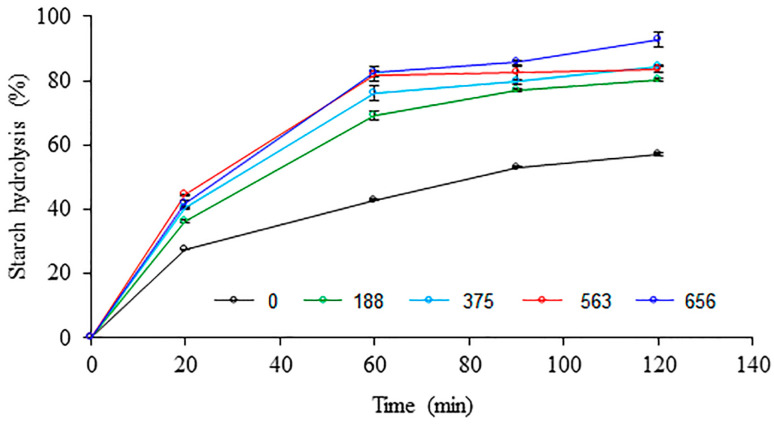
The percentage of starch hydrolysis during in vitro digestion of uncooked native and various PEF-treated wheat flours with different pulse numbers.

The starch hydrolysis rate of uncooked native and PEF-treated wheat flours is presented in Figure 8. The PEF-treated wheat flours had a higher starch hydrolysis rate than the untreated wheat flour. With increasing the specific energy input, the starch hydrolysis rate of PEF-treated wheat flours increased along the in vitro digestion period, which is related to CLSM and SEM results. The starch granules of PEF-treated wheat flours with mostly free surface proteins revealed by CLSM could be more susceptible to enzymatic hydrolysis. Moreover, some roughness or damage and some pores and cavities found on the surface of starch granules and smaller particle size after PEF treatment revealed by SEM and the particle size analyzer, respectively, could facilitate the enzyme penetration and accelerate the starch hydrolysis rate of PEF-treated wheat flours. The digestibility of starch granules was affected by the particle size distribution of starch granules [46]. Digestibility increased in small starch granules, e.g., rice starch, compared with large starch granules. The starch hydrolysis might be also related to the structural characteristics of starch granules in wheat flour. The destruction of the ordered structure of starch granules, as revealed by the reduced relative crystallinity of PEF-treated wheat flour, could enhance the starch digestibility. Based on the starch digestion rate, the starch fractions in terms of RDS, SDS, and RS are presented in Table 4. The untreated wheat flour had the lowest RDS and SDS content, but the highest RS content compared to PEF-treated wheat flours. The RDS and SDS contents tended to increase, while the RS content decreased with increasing the specific energy input. It meant that the PEF treatment of wheat flour at a high pulse number increased the susceptibility of starch to digestive enzymes, which was consistent with Duque et al. [8], who reported that PEF-treated oat flour at an elevated energy level had the highest RDS content, but lowest RS content.

## 4. Conclusions

The digestibility, particle size distribution, and physicochemical properties of wheat flour were found to be affected by PEF treatment at various specific energy inputs ranging from 0 to 656 kJ/kg. Most of the protein was removed or unfolded from the surface of the wheat starch granules, and the external and internal structures of the starch granules were damaged, with the extent of damage increasing with increasing specific energy input. The removal of protein in PEF-treated wheat flours facilitated the enzyme penetration and accelerated the starch hydrolysis. This resulted in a significant decrease in the overall viscosity and particle size of starch granules in wheat flour when the specific energy input was increased. The relative crystallinity of wheat flour decreased after PEF treatment, resulting in a significant decrease in gelatinization temperatures and enthalpy with increasing specific energy input. The use of PEF-treated wheat flour in various food products needs to be investigated for food application.

## Figures and Tables

**Table 1:** Pasting properties of native and various PEF-treated wheat flours.

Pulse Number	Specific Energy Input (kJ/kg)	Peak Viscosity	Trough	Breakdown	Final Viscosity	Setback	Pasting Temperature
		(mPa·s)	(mPa·s)	(mPa·s)	(mPa·s)	(mPa·s)	(°C)
0	0	440 ± 2 ^a^	280 ± 2 ^a^	160 ± 0 ^a^	730 ± 4 ^a^	450 ± 2 ^a^	81.01 ± 0.15 ^b^
200	94	431 ± 2 ^b^	275 ± 3 ^b^	156 ± 1 ^b^	690 ± 3 ^b^	429 ± 0 ^b^	81.20 ± 0.12 ^b^
400	188	415 ± 1 ^c^	268 ± 2 ^c^	147 ± 1 ^c^	675 ± 3 ^c^	421 ± 1 ^c^	81.12 ± 0.20 ^b^
600	282	390 ± 2 ^d^	249 ± 2 ^d^	141 ± 4 ^c^	647 ± 3 ^d^	398 ± 4 ^c^	81.20 ± 0.13 ^b^
800	375	364 ± 2 ^e^	241 ± 2 ^e^	123 ± 1 ^d^	631 ± 3 ^e^	390 ± 1 ^d^	82.10 ± 0.20 ^b^
1000	469	311 ± 2 ^g^	208 ± 3 ^f^	102 ± 2 ^e^	545 ± 3 ^g^	337 ± 1 ^f^	82.95 ± 0.10 ^a^
1200	563	320 ± 2 ^f^	215 ± 3 ^g^	105 ± 1 ^e^	568 ± 3 ^f^	353 ± 1 ^e^	82.85 ± 0.17 ^a^
1400	656	298 ± 1 ^h^	200 ± 3 ^h^	97 ± 2 ^f^	521 ± 2 ^g^	320 ± 5 ^g^	82.90 ± 0.05 ^a^

Means ± standard deviations (*n* = 3) within the same column followed by different superscript letters are significantly different (*p* ≤ 0.05).

**Table 2:** Thermal properties of native and various PEF-treated wheat flours.

Specific Energy Input (kJ/kg)	*T*_o_ (°C)	*T*_p_ (°C)	*T*_c_ (°C)	*T*_c_ − *T*_o_ (°C)	Enthalpy (kJ/kg)
0	59.19 ± 0.19 ^a^	64.67 ± 0.44 ^a^	70.50 ± 0.54 ^a^	11.31 ± 0.35 ^a^	4.24 ± 0.15 ^a^
188	58.74 ± 0.30 ^a^	63.84 ± 0.47 ^a^	70.01 ± 0.04 ^a^	11.27 ± 0.14 ^a^	3.26 ± 0.60 ^b^
375	57.95 ± 0.65 ^a^	62.05 ± 1.20 ^ab^	67.23 ± 0.04 ^b^	9.28 ± 0.61 ^b^	2.84 ± 0.03 ^c^
563	58.54 ± 0.45 ^a^	62.12 ± 0.26 ^b^	68.04 ± 0.46 ^b^	9.50 ± 0.01 ^b^	2.81 ± 0.00 ^c^
656	58.25 ± 0.08 ^a^	62.00 ± 0.10 ^b^	66.80 ± 0.40 ^c^	8.55 ± 0.32 ^c^	2.73 ± 0.00 ^d^

Means ± standard deviations (*n* = 3) within the same column followed by different superscript letters are significantly different (*p* ≤ 0.05).

**Table 3:** Crystalline and semi-crystalline lamellae structure parameters of PEF-treated wheat flour.

Electric Field Strength (kJ/kg)	1047/1022	995/1022
0	0.744 ± 0.012 ^a^	1.017 ± 0.004 ^a^
188	0.729 ± 0.002 ^b^	1.015 ± 0.008 ^ab^
281	0.719 ± 0.008 ^b^	1.013 ± 0.012 ^b^
375	0.715 ± 0.004 ^b^	1.012 ± 0.003 ^b^
469	0.710 ± 0.010 ^c^	1.010 ± 0.002 ^c^
656	0.708 ± 0.004 ^c^	1.009 ± 0.005 ^c^

Means ± standard deviations (*n* = 3) within the same column followed by different superscript letters are significantly different (*p* ≤ 0.05).

**Table 4:** RDS, SDS, and RS content of uncooked native and various PEF-treated wheat flours with different specific energy inputs.

Specific Energy Input (kJ/kg)	RDS (%)	SDS (%)	RS (%)
0	27.59 ± 0.12 ^e^	29.68 ± 0.48 ^d^	42.73 ± 0.42 ^a^
188	36.51 ± 0.48 ^d^	43.74 ± 0.45 ^b^	9.75 ± 0.36 ^b^
375	40.50 ± 0.52 ^c^	44.13 ± 0.42 ^b^	15.37 ± 0.27 ^c^
563	44.56 ± 0.25 ^a^	39.08 ± 1.03 ^c^	16.36 ± 0.86 ^c^
656	41.72 ± 1.12 ^b^	51.00 ± 2.50 ^a^	7.28 ± 2.43 ^d^

Values are given as means of triplicate determinations ± standard deviation. Values in the same column with different superscripts are significantly different (*p* ≤ 0.05). RDS, rapidly digestible starch; SDS, slowly digestible starch; RS, resistant starch.

## Data Availability

Data is contained within the article.
